# Ascorbic acid metabolism during sweet cherry (*Prunus avium*) fruit development

**DOI:** 10.1371/journal.pone.0172818

**Published:** 2017-02-28

**Authors:** Dong Liang, Tingting Zhu, Zhiyou Ni, Lijin Lin, Yi Tang, Zhihui Wang, Xun Wang, Jin Wang, Xiulan Lv, Hui Xia

**Affiliations:** 1 Institute of Pomology and Olericulture, Sichuan Agricultural University, Chengdu, Sichuan, China; 2 College of Horticulture, Sichuan Agricultural University, Chengdu, Sichuan, China; University of Tsukuba, JAPAN

## Abstract

To elucidate metabolism of ascorbic acid (AsA) in sweet cherry fruit (*Prunus avium* ‘Hongdeng’), we quantified AsA concentration, cloned sequences involved in AsA metabolism and investigated their mRNA expression levels, and determined the activity levels of selected enzymes during fruit development and maturation. We found that AsA concentration was highest at the petal-fall period (0 days after anthesis) and decreased progressively during ripening, but with a slight increase at maturity. AsA did nevertheless continue to accumulate over time because of the increase in fruit fresh weight. Full-length cDNAs of 10 genes involved in the L-galactose pathway of AsA biosynthesis and 10 involved in recycling were obtained. Gene expression patterns of GDP-L-galactose phosphorylase (*GGP2*), L-galactono-1, 4-lactone dehydrogenase (*GalLDH*), ascorbate peroxidase (*APX3*), ascorbate oxidase (*AO2*), glutathione reductase (*GR1*), and dehydroascorbate reductase (*DHAR1*) were in accordance with the AsA concentration pattern during fruit development, indicating that genes involved in ascorbic acid biosynthesis, degradation, and recycling worked in concert to regulate ascorbic acid accumulation in sweet cherry fruit.

## Introduction

Ascorbic acid (AsA) has several essential functions in plant physiology. AsA is the most abundant water-soluble antioxidant in higher plants, participates in the detoxification of reactive oxygen species, and has an important role in promoting resistance to senescence [1] and numerous environmental stresses, such as ozone [2, 3], dehydration stress [4, 5], high light [6] and salt stress [7]. Also, AsA operates as a cofactor and take part in the regulation of some fundamental cellular processes (e.g. photoprotection, the cell cycle and cell expansion) and biosynthesis of important plant hormones (e.g. including ethylene, jasmonic acid, salicylic acid, abscissic acid gibberellic acid) [8, 9]. Moreover, fruit and vegetables are a primary source of dietary intake of vitamin C for humans, because primates and some other animals lack the ability to synthesize AsA [10].

**Fig 1:**
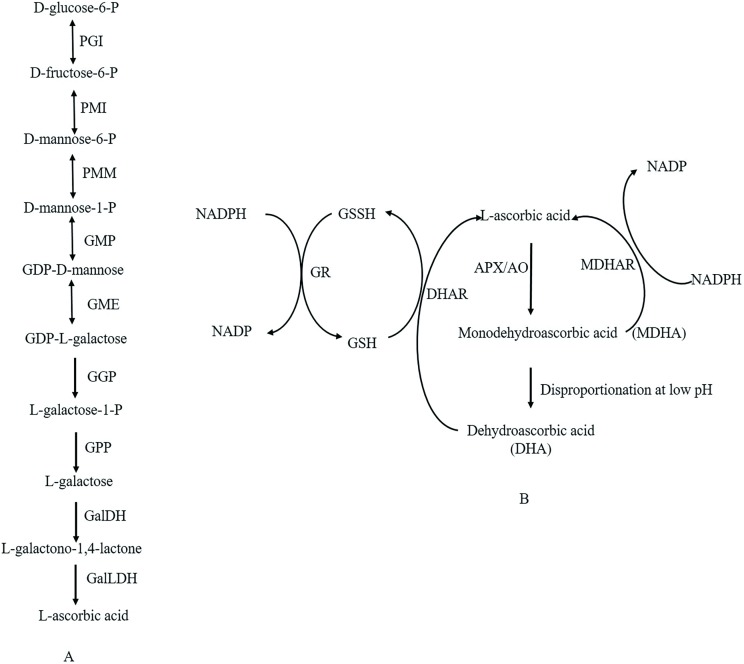
**L-ascorbic acid biosynthesis by the L-galactose pathway (A) and the ascorbate-glutathione cycle (B) in plants.** The enzymes catalyzing the reactions are as follows: glucose-6-phosphate isomerase (GPI), mannose-6-phosphate isomerase (PMI), phosphomannomutase (PMM), GDP-mannose pyrophosphorylase (GMP), GDP-mannose-3′,5′-epimerase (GME), GDP-L-galactose phosphorylase (GGP), L-galactose-1-phosphate phosphatase (GPP), L-galactose dehydrogenase (GalDH), L-galactono-1,4-lactone dehydrogenase (GalLDH), ascorbate peroxidase (APX), monodehydroascorbate reductase (MDHAR), dehydroascorbate reductase (DHAR), ascorbate oxidase (AO), and glutathione reductase (GR).

Because of these unique functions, as well as its benefits to human health, mounting attention has been paid to AsA metabolism and regulation in plant tissues. *De novo* biosynthesis is believed to be the main reason for its accumulation in plant. At least four AsA synthesis pathways have been proposed in plants according to the molecules acting as precursors: L-galactose [11], L-glucose [12], D-galacturonic acid [13] and myo-inositol pathways [14]. Among them, the L-galactose pathway has been deemed to be the main route for AsA accumulation in different species [15, 16], and all structural genes from this pathway have been obtained in various species [17–20]. Briefly, the L-galactose pathway consists of a series of successive reactions (Fig 1A): starting from D-glucose-6P, then D-mannose-1-phosphate, guanosine diphosphate-D-mannose (GDP-D-mannose), GDP-L-galactose, L-galactose and L-galactone-1,4-lactone, which are intermediates to the formation of AsA [19, 20].

The AsA levels in plants are maintained by an efficient balance among biosynthesis, oxidation and recycling, known as the AsA–GSH cycle [9] (Fig 1B). The first step of the pathway is the detoxification of reactive oxygen species by ascorbate peroxidase (APX) and ascorbate oxidase (AO) catalyzed peroxidation of AsA which generates monodehydroascorbate (MDHA). MDHA is either reduced back to AsA by monodehydroascorbate reductase (MDHAR) or it undergoes non-enzymatic disproportionation to AsA and dehydroascorbate (DHA). The DHA molecules are reduced to AsA by dehydroascorbate reductase (DHAR) using GSH as the reductant. GSH is regenerated from the oxidized glutathione dimmers (GSSG) by NADPH-dependent Glutathione oxidoreductase (GR).

Sweet cherry (*Prunus avium* L.) is one of the popular and economically-valuable fruit cultivated in temperate regions of the world and is recognized for its nutraceutical properties and antioxidant activity. Although transcriptional regulation of AsA biosynthesis has been studied extensively in several horticultural crops, such as peach [21], kiwifruit [17], apple [18], strawberry [22], chestnut [20] and Chinese jujube [23], little is known about AsA metabolism in sweet cherry. To gain new insight into that process in sweet cherry fruit, we identified a complete and continuous set of sequences involved in the L-galactose pathway from sweet cherry fruit. Moreover, we systematically investigated the accumulation of AsA, mRNA expression of genes and key enzyme activity involved in its biosynthesis as well as recycling during fruit development. Our objective was to provide useful information to improve AsA concentration in sweet cherry fruit and to explore the mechanisms that regulate AsA accumulation in cells.

## Materials and methods

### Plant materials

Eight-year-old cherry (*P*. *avium* ‘Hongdeng’) trees were grown at a 3m × 4m spacing in an orchard in Hanyuan County, Sichuan Province, China (29.51°N, 102.62°E). There were no specific permissions required for local activities in experimental field, and the field studies did not involve endangered or protected species. Fruit was harvested at 10 ± 1d intervals following anthesis. The day on which the petals had just dropped off was designated as 0 days after anthesis (DAA). Fruit were immediately frozen in liquid nitrogen and stored at −80°C. On each collection date, at least fifteen fruits were harvested from each of five trees, and each three fruits represented one of five replications. At least 15 young, mature and old leaves were collected respectively.

### Assays for AsA

Frozen tissue (2.0g) was added to extract AsA and determined essentially as described in [20, 23] by a high-performance liquid chromatography (HPLC). To determine the total AsA (T-AsA) level, the method described by [17, 18] was used. Dehydroascorbic acid (DHA) content was evaluated as the difference between total and reduced AsA levels.

HPLC was carried out using Agilent 1260 with a VWD detector, SB-Zobax C18 column (150×4.6mm, 5μm). The mobile phase was composed of 15% methanol and 85% metaphosphoric acid aqueous solution, pH 2.5. The flux was set to 1ml/min and the injection volume was 10μl. The temperature of the column was set at 35°C; the detection wavelength was set at 243nm.

### Cloning of cDNAs encoding enzymes involved in AsA biosynthesis and recycling

Standard primers for gene cloning were designed according to the unigene sequence obtained from our transcriptome data (unpublished) and BLAST searching in the GenBank Prunus EST results (Table 1).

**Table 1:** Standard primers used for gene cloning.

Gene	Forward primer (5′-3′)	Reverse primer (5′-3′)
*GPI*	GGCACATACATGGTGTGAGG	TCCTAAATGCCGGAACATAA
*PMI*	TGAAGCACCACAATCAAAGG	TGCTTGCACTAAAGCCAAGA
*PMM*	CCATTTCGGAGCTCTCATTC	CCGAAACCAAAAAGTTGGAA
*GMP*	GGAAAGCAAAGGGCATATCA	CAGCCTGAATCCAGAGAAGC
*GME*	GCCTTGTTTCCTTTGCTCTG	CTGAGCAATGCTTCAGATGG
*GGP1*	TCCCTCCGATCTTACCTTCC	TTGCTGAGCGTCTATTTCCA
*GGP2*	CATCACGGCTATACACGGAG	ATAAACCCAAATGGCAAACT
*GPP*	CCGCATTTCCTTCTCTCATC	AGCTTCGAGCAAGATTACCG
*GalDH*	ATATTTTTCGCTTCACTTCC	ATTTTATTGATTCATCTCCA
*GalLDH*	AAAGAGGGAAGGAGGAGCAG	TGAAGCCAAAATGCATTCAA
*APX1*	TCCTTCCCTCCAAACAACAC	CACACATAGGCAACCAATGC
*APX2*	CTACCCAAACAGAGGGTGGA	AGCCATCGCTGCCTTATTTA
*APX3*	AATGGCGTTCTCCTTCCTTT	AGCTATCCGTGGGTCAAACA
*AO1*	TGTCTCTTTCTGTAACCTTC	TTCTTTCTTTTTCCTCTCTC
*AO2*	TTTTAACTTGTTGTAGTTTG	ATAGTTGAATGGTATGAGAT
*DHAR1*	CTGGCACTCTTCCTCACTTC	CTTCCTTTTTTTGGCTATCT
*DHAR2*	ACACCGACCAATGCGTTAAT	TCCACATCTGACATGCACAC
*GR1*	TCATGGCCACCTCTCTCTCT	CATGCTCTGCAGAACCTCAA
*GR2*	GCTTCGCCATCTTCAACTTC	CGTCCTATTGCTTGCATCCT
*MDHAR*	GCCAGCCACTGTCCTTAAAC	TGCAAAGGAGACAGCTTCAA

Total RNA was extracted from samples by the modified CTAB method [24], and then reverse-transcription PCR was used to amplify whole cDNA sequences for every gene. The obtained fragments were cloned into pMD19-T vector (Takara) and unique clones of positive transformants were sequenced. Homology comparison was performed using the BLAST program in NCBI (http://www.ncbi.nlm.nih.gov).

### mRNA expression analyses

Total RNA was extracted from samples by the modified CTAB method [24], and DNase was used to clean DNA before reverse-transcription reaction. Gene-specific primers were designed from the sequences that we had cloned using Primer3 online software (Table 2).

**Table 2:** Standard primers used for mRNA expression.

Gene	Forward primer (5′-3′)	Reverse primer (5′-3′)
*GPI*	TTGACAATACCGATCCAGCA	GTTATCGCGACACCCTGTTT
*PMI*	TTCAAAAATGGGGCTCTGAC	CCTTATCTGGGTGTGCCTGT
*PMM*	GTTCCGAAGTGGGATGCTTA	TTCTCGCGAAGGATGGATAC
*GMP*	TGGCATCAAGATCACATGCT	AGCTTCTCCTCCATGGGATT
*GME*	GCTTCATCCAGTCCAACCAT	GGCATCAGACTCCTTCAAGC
*GGP1*	TTCTAGCGCAGTGGGAAGAT	TTTTGTTCTTGCCCAGCTTT
*GGP2*	GCCTGTGAAACCAAGGTGAT	GCCCAACTTTGGTGAGGTTA
*GPP*	GTACGTGGAGGAGGTGCATT	AGCCACTCATGCGAAGAGAT
*GalDH*	CCAGCATCTGCTGAATTGAA	CAGCAGCAATGTTCTCCTCA
*GalLDH*	ACTCCTTCCCTTTCCCTGAA	TTTTCTTCTCATGGGCATCC
*APX1*	GAGCCAAATTTGATCCTCCA	CTTATCTGGGCTTCCACCAA
*APX2*	CGGATCATTTGAGGGATGTC	ACGTTCCTTGTGGCACCTAC
*APX3*	AATGGCGTTCTCCTTCCTTT	GTGGCACCAACACATTTGAG
*AO1*	TGGTGGGGTTTTTAAAGCTG	ATCCATTCCAACAACCCAAA
*AO2*	TCCAGAATTGGGAAGACACC	ACATTGTCCAGTGCCACGTA
*DHAR1*	ATGGGAAGGAGGTTTCTGCT	TGGAAGGGAATCTGGAACTG
*DHAR2*	ATGGGTCAGAACAGGCTTTG	ATCTGCGGCAGTGATCTTCT
*GR1*	GAAAGCTGGCTTGACAAAGG	CAACCACGATCAAACACCAG
*GR2*	CCATAGGCGTGGAACTTGAT	CCTCCATTAAAGCCACAGGA
*MDHAR*	CAAGCTCAACACCGCTTACA	ATTGATGGAATGCTCGGAAG
*TEF2*	GGTGTGACGATGAAGAGTGATG	TGAAGGAGAGGGAAGGTGAAAG
*ACT*	CTTGCATCCCTCAGCACCTT	TCCTGTGGACAATGGATGGA

Expression of the genes involved in AsA biosynthesis and recycling was evaluated by quantitative reverse transcription PCR (qRT-PCR). qRT-PCR was performed with a PrimeScript™ RT Reagent Kit with gDNA Eraser (Perfect Real Time) (Takara) and a SYBR Premix Ex Taq kit (Takara). The amplified PCR products were quantified by a CFX96 Touch™ Real-Time PCR Detection Systems (Bio-Rad). qRT-PCR experiments were done with four technical replications. All data were analyzed by the 2^-△△CT^ method, with CFX Manager™ Software. *Translation elongation factor 2* (*TEF2*) and *Actin 7* /*actin 2* (*ACT*) were used as reference genes for the relative quantification of PCR products (Table 1). The expression of these reference genes has been shown to be stable during cherry fruit development [25].

### Assays of GalLDH and GalDH activities

GalLDH (EC 1.3.2.3) and GalDH (EC 1.1.1.117) enzyme activity were prepared and detected according to the method of [17, 20]. The GalLDH activity was determined by cytochrome c was reduced. One unit of activity was defined as the reduction of 1mmol of cytochrome c per minute. Activity of GalDH was calculated in terms of mmol of NAD^**+**^ reduced per minute.

### Assays of APX, GR, DHAR, and MDHAR activities

For extraction of enzyme solution, samples were homogenized with 8ml of 50mM potassium phosphate buffer (pH 7.5) containing 1mM EDTA, 1mM DTT, 0.3% (v/v) Triton X-100, 2% (w/v) PVP and 2% (w/v) mercaptoethanol. The homogenates were centrifuged at 16,000g for 20min at 4°C and the supernatants were collected for enzyme assays.

APX (EC 1.11.1.11) activity was assayed by measuring the decrease in AsA concentration at 290nm according to Nakano and Asada [26] with slight modifications. GR (EC 1.6.4.2) activity was calculated by measuring the decrease in absorbance at 340nm due to oxidation of NADPH as described by Ma and Cheng [27]. DHAR (EC 1.8.5.1) and MDHAR (EC 1.6.5.4) activities were assayed using the method of Ma and Cheng [27]. DHAR activity was expressed as μmol of DHA reduced per minute. MDHAR activity was calculated in terms of mmol of NADH oxidized per minute.

### Statistical analysis

ANOVA was performed using SPSS software (SPSS Inc., Chicago, IL, USA). Each treatment was replicated five times. Results were represented as the means ± standard deviation (SD). Significant differences were detected using Duncan's test at *P*<0.05 level.

## Results

### Changes in AsA levels during cherry fruit development

**Fig 2:**
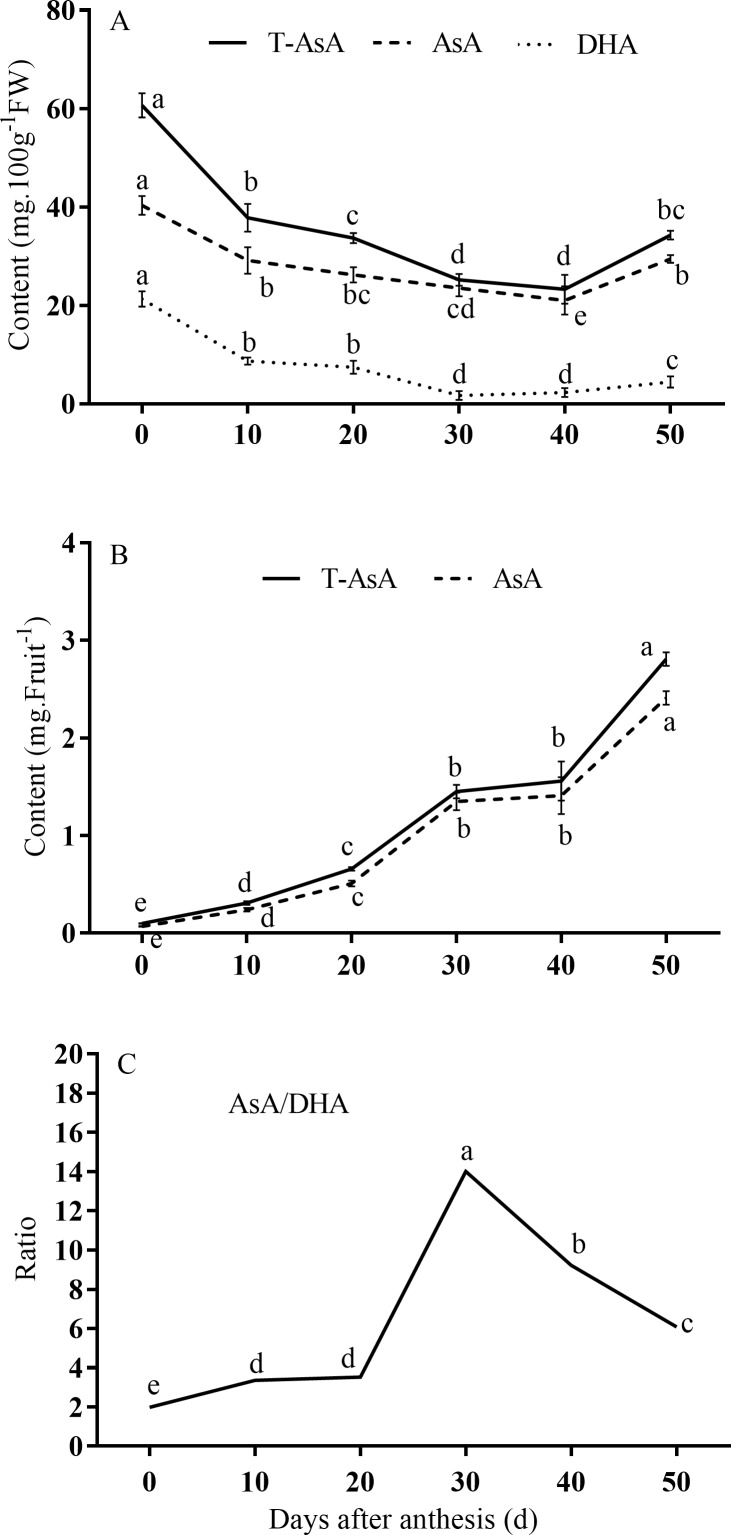
Changes in ascorbic acid (AsA) accumulation levels during fruit development. (A) Total AsA (T-AsA), AsA, and dehydroascorbate (DHA) concentrations based on fresh weight of fruit; (B) T-AsA and AsA content per fruit; (C) AsA/DHA ratio. T-AsA and AsA content per fruit was obtained by multiplying average fruit weight by concentration based on fresh weight.

T-AsA and AsA levels for ‘Hongdeng’ cherry were monitored from the young fruit stage (0 DAA) up to maturity. Based on unit fresh weights, fruits at 0 DAA had the highest concentrations of T-AsA, DHA, and AsA, but these levels decreased clearly until 40 DAA, and then increased slightly to 50 DAA (Fig 2A, S1 Appendix). However, T-AsA and AsA accumulation per fruit generally increased throughout. Content levels of T-AsA and AsA were lowest at 0 DAA and then increased rapidly to 30 DAA before a relatively stable period (30–40 DAA), and then increased again from 40–50 DAA (Fig 2B, S1 Appendix). Reduced AsA concentration was lower than that of oxidized AsA during whole fruit ripening process. The AsA/DHA ratio remained at a lower level from 0–20 DAA, increased rapidly from 20–40 DAA, and then dramatically decreased from 40–50 DAA (Fig 2C, S1 Appendix).

### cDNA isolation of genes involved in AsA biosynthesis and recycling in cherry fruit

Since there were no sequences involved in AsA metabolism of sweet cheery available in GenBank, we cloned sequences involved in AsA synthesis and recycling based on unigene sequences from a transcriptome database we had sequenced. The cDNA sequences of a total of 20 genes were identified; ten of them encoded proteins that belonged to the L-galactose pathway of AsA synthesis *(PacGPI*, *PacPMI*, *PacPMM*, *PacGMP*, *PacGME*, *PacGGP1*, *PacGGP2*, *PacGPP*, *PacGalDH*, and *PacGalLDH)*, and the other ten genes encoded enzymes that were involved in the AsA-GSH cycle *(PacAO1*, *PacAO2*, *PacAPX1*, *PacAPX2*, *PacAPX3*, *PacDHAR1*, *PacDHAR2*, *PacMDHAR*, *PacGR1*, and *PacGR2)*. The cDNAs for all these genes contained the entire protein-encoding regions. NCBI homology searches for these cloned sequences showed high degrees of similarity to the expected biosynthetic enzymes in *Prunus mume*, *Prunus persica*, and *Pyrus* × *bretschneideri* (Table 3). We concluded that the cloned sequences were cherry orthologs that encoded AsA biosynthetic and recycling enzymes and could therefore be used for mRNA expression analyses.

**Table 3:** Genbank homology search results for cloned sequences in this study.

Accession No. in Genbank	Gene	ORF (bp)	Top hit ortholog			
			Accession No.	Organism	Annotation	Identities
KX196283	*GPI*	1707	XM_009366209.1	*Pyrus* × *bretschneideri*	glucose-6-phosphate isomerase	92%
KX196284	*PMI*	1323	XM_008246293.1	*Prunus mume*	mannose-6-phosphate isomerase	98%
KX196285	*PMM*	744	KC339527.1	*Prunus persica*	phosphomannose mutase	98%
KX196286	*GMP*	1086	AB457581.1	*Prunus persica*	GDP-D-mannose pyrophosphorylase	99%
KX196287	*GME*	1131	AB457582.1	*Prunus persica*	GDP-D-mannose-3′,5′-epimerase	98%
KX196288	*GGP1*	1107	XM_008227382.1	*Prunus mume*	GDP-L-galactose phosphorylase	98%
KX196289	*GGP2*	1341	XM_008228760.1	*Prunus mume*	GDP-L-galactose phosphorylase	98%
KX196290	*GPP*	813	AB457584.1	*Prunus persica*	L-galactose-1-phosphate phosphatase	99%
KX196291	*GalDH*	975	AB457585.1	*Prunus persica*	L-galactose dehydrogenase	97%
KX196292	*GalLDH*	1791	XM_008242509.1	*Prunus mume*	L-galactono-1,4-lactone dehydrogenase	98%
KX196297	*APX1*	1363	XM_008221397.1	*Prunus mume*	L-ascorbate peroxidase	97%
KX196298	*APX2*	753	XM_008240917.1	*Prunus mume*	L-ascorbate peroxidase	98%
KX196293	*APX3*	1050	XM_008221119.1	*Prunus mume*	L-ascorbate peroxidase	97%
KX196295	*AO1*	1611	XM_008237876.1	*Prunus mume*	L-ascorbate oxidase	98%
KX196296	*AO2*	1632	XM_008226470.1	*Prunus mume*	L-ascorbate oxidase	100%
KX196299	*DHAR1*	801	XM_008230975.1	*Prunus mume*	dehydroascorbate reductase	98%
KX196300	*DHAR2*	639	XM_008235328.1	*Prunus mume*	dehydroascorbate reductase	100%
KX196294	*GR1*	1674	XM_008237191.1	*Prunus mume*	glutathione reductase	96%
KX196301	*GR2*	1491	XM_008226379.1	*Prunus mume*	glutathione reductase	99%
KX196302	*MDHAR*	849	XM_008229040.1	*Prunus mume*	monodehydroascorbate reductase	98%

### Changes in mRNA expression of genes involved in AsA synthesis, degradation, and recycling during fruit development

**Fig 3:**
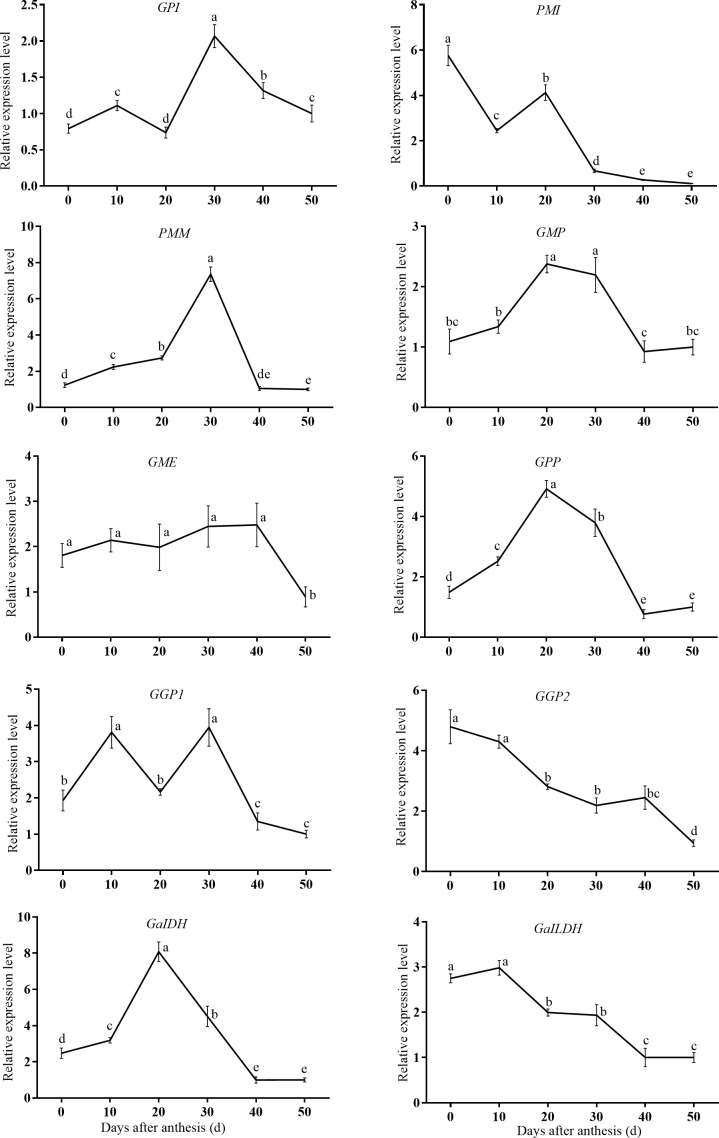
Changes in relative mRNA expression for genes encoding enzymes involved in the L-galactose pathway of ascorbic acid synthesis, including *GP1*, *PMI*, *PMM*, *GMP*, *GME*, *GPP*, *GGP1*, *GGP2*, *GalDH*, and *GalLDH*.

**Fig 4:**
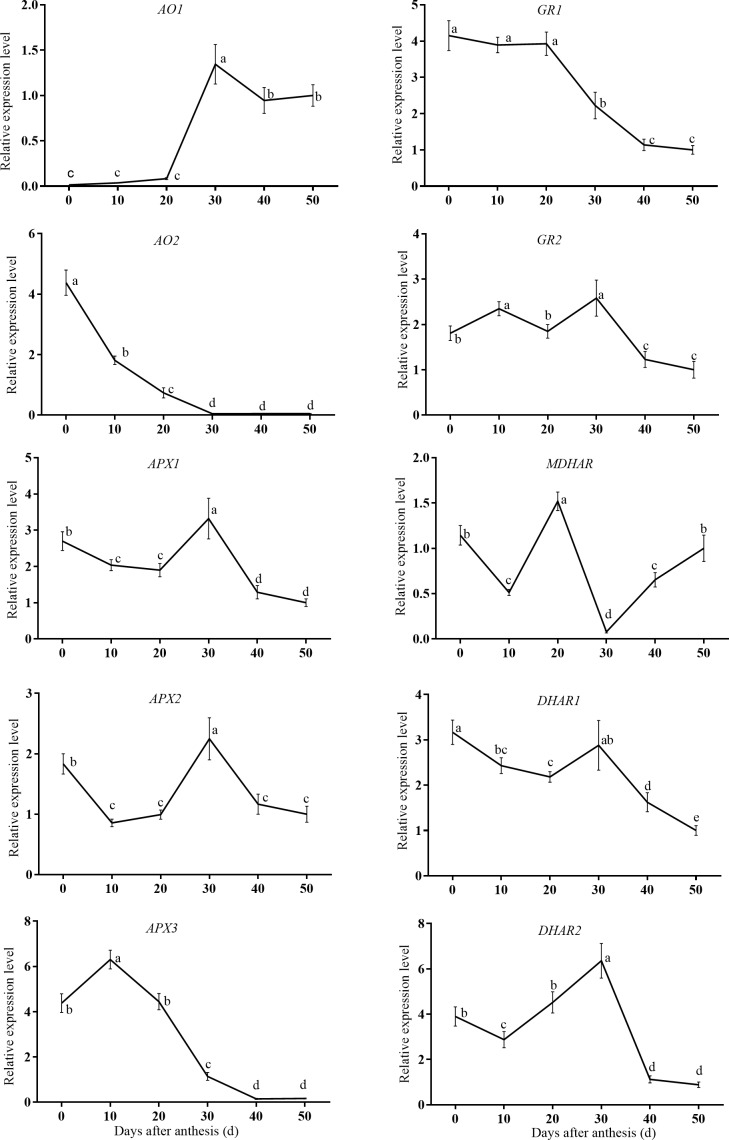
Changes in relative mRNA expression for genes encoding enzymes involved in ascorbic acid degradation and recycling during fruit development, including *AO1*, *AO2*, *APX1*, *APX2*, *APX3*, *GR1*, *GR2*, *MDHAR*, *DHAR1*, and *DHAR2*.

To understand the transcriptional regulation of genes involved in AsA accumulation in sweet cherry fruits, the expression of the genes encoding specific enzymes in the main AsA biosynthetic (Fig 3), degradation, and recycling pathways (Fig 4) during cherry fruit development were investigated using qRT-PCR.

The genes exhibited varying expression patterns. In the L-galactose pathway, *GPI*, *PMM*, *GMP*, *GPP*, and *GalDH* were expressed at relatively low levels at the young fruit stage; they increased to their highest levels during the enlargement stage (20 DAA or 30 DAA), and then decreased towards maturation. By contrast, the relative expression of *GME* remained steady before 40 DAA and then clearly declined until 50 DAA. And *GGP1* displayed an “up-down-up-down” expression pattern during the whole course of development. *PMI*, *GGP2*, and *GalLDH* showed a similar expression pattern, with levels at their highest at 0 DAA, and then decreasing persistently with fruit ripening (S2 Appendix).

In the AsA-GSH cycle, the expression levels of *APX1* and *APX2* decreased during the young fruit stage, increased to their maximum at 30 DAA, and then decreased towards maturation. The relative expression of *APX3*, however, peaked at 10 DAA and then clearly declined to a steady and lower level after 40 DAA. *DHAR2* displayed a similar expression profile to *APX1* and *APX2*. Relative expression of AO1 mRNA showed no obvious changes from 0–20 DAA but clearly increased by 30 DAA, after which it remained at a constant level until fruit maturation. Moreover, the relative expression of *AO2*, *GR1*, and *DHAR1* mRNA had its highest peak in 0 DAA fruit followed by a marked decline, and then it remained at a stable and very low level. The mRNA expression pattern of *MDHAR* was complicated, with three peaks (0, 20, and 50 DAA) and two troughs (10 and 30 DAA) (Fig 4), while *GR2* showed an “up-down-up-down” pattern (S2 Appendix).

### Expression of genes involved in AsA biosynthesis and recycling in leaves

**Fig 5:**
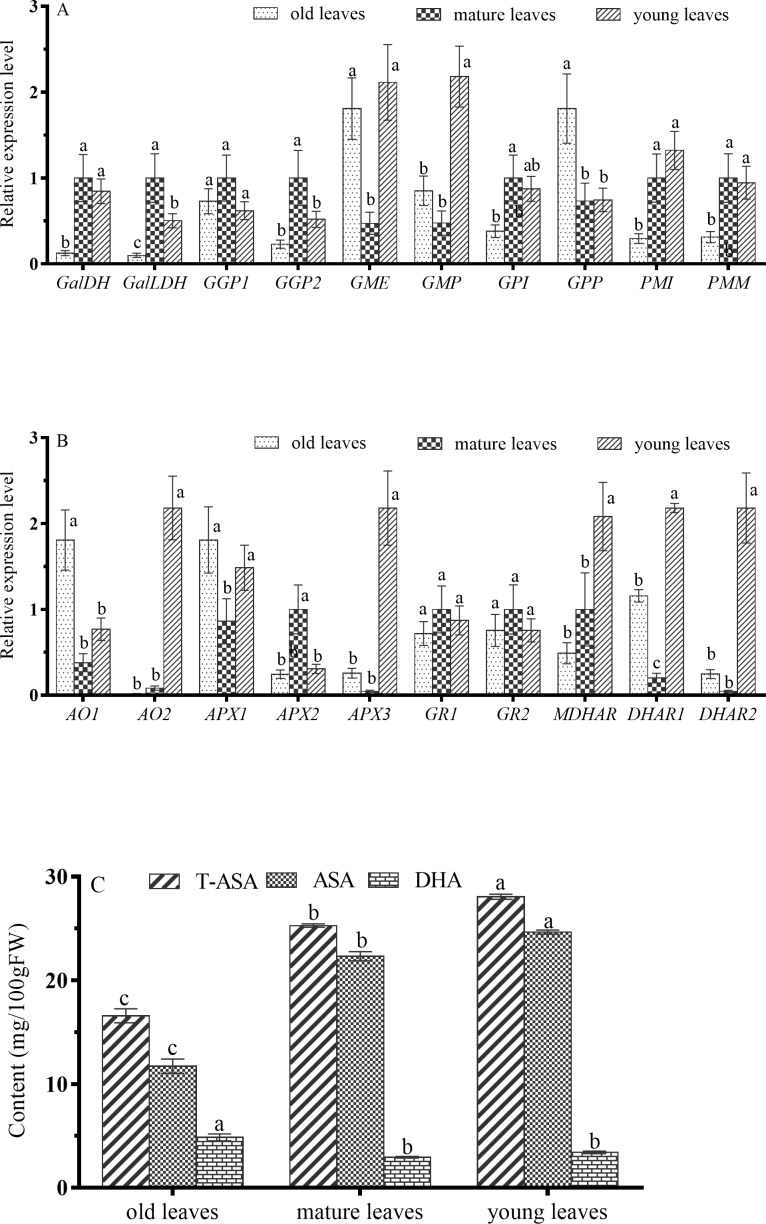
Changes in relative mRNA expression for genes encoding enzymes involved in ascorbic acid synthesis and recycling in young, mature, and old leaves. (A) *GPI*, *PMI*, *PMM*, *GMP*, *GME*, *GGP1*, *GGP2*, *GPP*, *GalDH*, and *GalLDH*; (B) *AO1*, *AO2*, *APX1*, *APX2*, *APX3*, *DHAR1*, *DHAR2*, *MDHAR*, *GR1*, and *GR2*; (C) ascorbic acid (AsA) accumulation levels in old leaves, mature leaves, and young leaves.

To explore AsA metabolism in leaves of sweet cherry, we analyzed the expression of genes related to AsA metabolism in young leaves, mature leaves, and old leaves, and we also measured reduced and oxidized AsA concentrations (Fig 5).

AsA concentrations in leaves ranged from 16.5 to 28mg per 100g FW (Fig 5C), lower than the average AsA level in fruit. T-AsA and AsA both were highest in young leaves, significantly higher than they were in mature and old leaves. Accordingly, old leaves had lowest T-AsA and AsA concentrations, but the highest DHA concentrations.

Six of the genes related to AsA biosynthesis, *GalDH*, *GalLDH*, *GGP1*, *GGP2*, *GPI*, and *PMM*, had their highest expression levels in mature rather than young or old leaves, while three genes, *GME*, *GMP*, and *PMI*, had their highest levels of transcription in young leaves (Fig 5A). Five genes involved in degradation and recycling, *AO2*, *APX3*, *MDHAR*, *DHAR1*, and *DHAR2*, had extremely high expression levels in young leaves in contrast to mature and old leaves (Fig 5B). *GR1* and *GR2* expression levels did not significantly differ in young, mature, and old leaves. Considered together, these results indicate that high AsA concentrations in young leaves can be attributed to the high transcription levels of genes involved in AsA recycling (S3 Appendix).

### Changes in activities of key enzymes involved in AsA synthesis and recycling during fruit development

**Fig 6:**
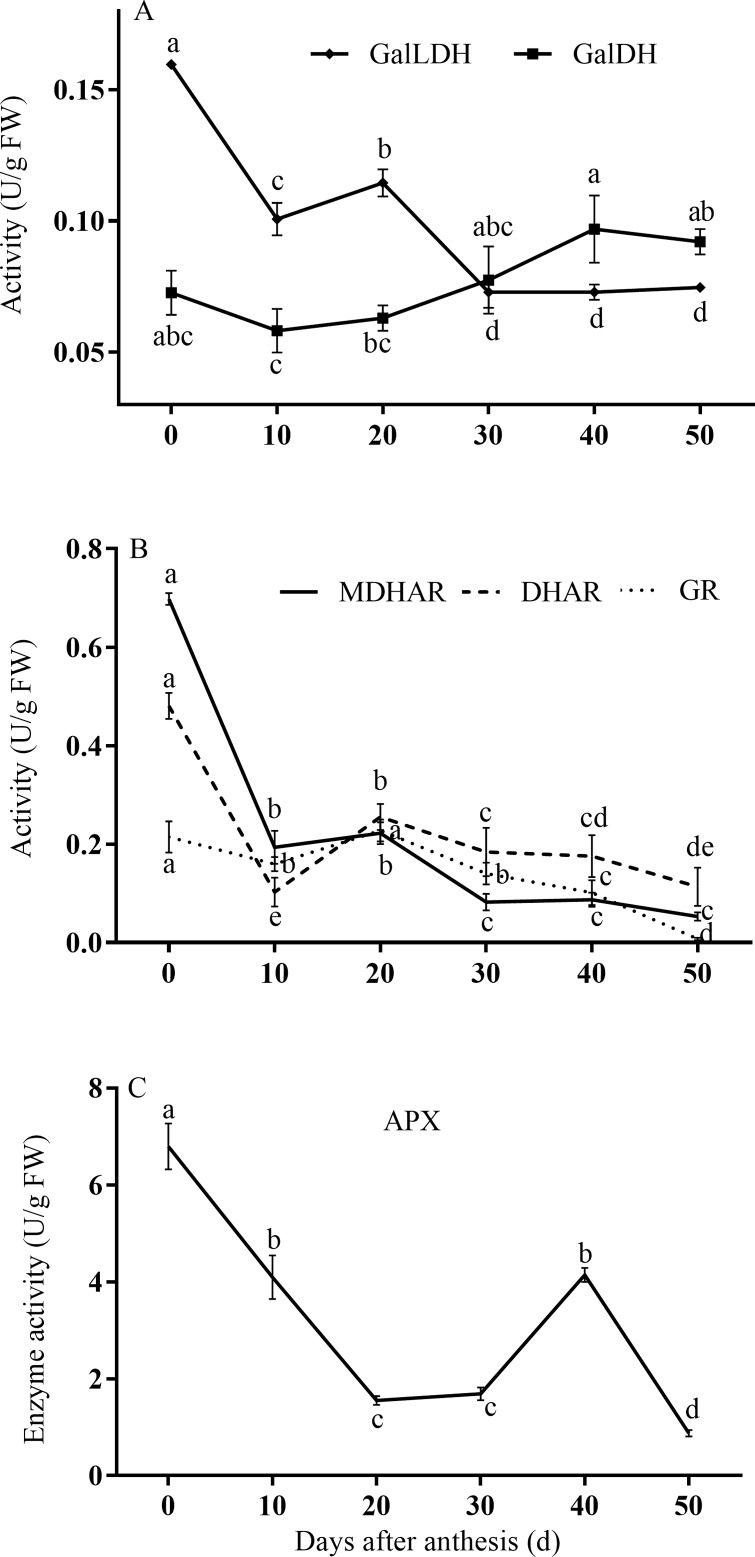
**Changes in enzyme activity levels of (A) GalLDH and GalDH; (B) MDHAR, DHAR, and GR; (C) APX, based on fresh weight during fruit development**.

We selected several important enzymes involved in AsA metabolism for evaluation (Fig 6). On a fresh-weight basis, the patterns of GalLDH activity decreased gradually during fruit development and ripening. By contrast, GalDH activity increased slowly until fruit maturation (Fig 6A).

The activity of MDHAR peaked in 0 DAA fruit, and then clearly declined by 10 DAA. After that time point, MDHAR activity slightly decreased until maturity. Compared with its maximum in 0 DAA fruit, DHAR activity declined to its lowest point at 10 DAA; it increased from 10–20 DAA, and then declined slowly through to maturity. From 0–20 DAA, GR activity remained at a high and stable level, but clearly declined from 20 DAA to fruit maturation (Fig 6B). APX activity decreased significantly from 0–20 DAA but remained nearly constant and at a low level until maturity (Fig 6C).

## Discussion

### AsA accumulation during fruit development

AsA levels in plant cells vary between species and even between genotypes of a given species, and they are highly regulated by developmental processes [28]. In the present study, the AsA levels in sweet cherry fruit as determined by HPLC were found to be highest during the petal-fall period, they declined progressively during development, but then slightly increased at the last stage. Similar patterns have been seen in results from peach [21] and apple [18]. By contrast, AsA levels tend to increase over time in the fruit of grapes [29], strawberry [22], and chestnut rose [20]. In other fruits, such as kiwifruit and blueberry [30, 17], AsA concentration increases rapidly in the early stage, but declines in later stages. Variation in the AsA content trend during maturation determines the final AsA level in the fruit.

### AsA biosynthesis and related gene expression during fruit development

AsA biosynthesis in situ is considered to be the prime mechanism leading to AsA accumulation in most plants, including blackcurrant [31], peach [21], kiwifruit [17], apple [18], and strawberry [22]. In the present study, we obtained 10 cDNA sequences involved in the L-galactose pathway. Results of qRT-PCR demonstrated that 10 key genes involved in AsA synthesis through the L-galactose pathway were expressed during sweet cherry fruit development (Fig 3). It is therefore understandable that genes/enzymes involved in the L-galactose pathway exist in sweet cherry fruit cells and may play key roles in AsA biosynthesis. Among these genes, *GGP2* and *GalLDH* exhibited expression pattern that were in accord with the AsA levels during sweet cherry fruit ripening, indicating their key regulatory role in AsA biosynthesis. *GGP* has been supposed to be the key regulatory gene of the L-galactose pathway for AsA biosynthesis in kiwifruit [32] and apple [33]. The expression of *GalLDH* mostly correlated with the respective AsA content levels in different *Myrciaria dubia* tissues [34].

### Expression patterns of genes involved in AsA degradation and recycling during fruit development

AsA concentration is determined not only by its biosynthesis but also by its degradation and recycling. We examined the pattern of expression of five genes involved in the degradation pathways (three *APX* and two *AO* isoforms) and five genes involved in AsA recycling during fruit maturation. *AO2* and *APX3* exhibited high expression levels at the beginning, and then decreased persistently during fruit development, a pattern in accordance with the observed changes in AsA levels. The high levels of *AO* transcripts during early fruit development may participate in cell growth [35]. The rapid decline of *APX3* expression abundance in the fruit enlargement stage, simultaneously with fall of AsA, suggests that it plays an important role in AsA degradation.

With respect to the enzymes involved in recycling, MDHAR activity was found to be correlated with AsA concentration, but expression data showed that the *GR1* and *DHAR1* expression patterns tracked the AsA concentration more closely than did *MDHAR*. A high correlation between enzyme activity and *MDHAR* and *DHAR* mRNA abundance in response to ripening and stresses has been observed in acerola fruits [36]. Correlations between the expression and activity levels of *DHAR* and the level of AsA have been observed in kiwifruit [17] and chestnut rose [20]. The variation in strawberry fruit AsA content also correlated well with *MDHAR* [22].

The recycling and degradation steps, in particular AO2, APX3, GR1, and DGAR1, therefore appear to be important factors in the regulation of AsA content in the fruit of sweet cherry.

### Expression of genes involved in AsA biosynthesis and recycling in leaves

Our results showed that sweet cherry had highest AsA content in young leaves, followed by a continuous decline with increasing leaf age. Whereas old leaves had highest DHA content compared with young and mature young leaves (Fig 5C). These results indicate that the rate of AsA synthesis exceeds the rate of oxidation loss in young leave, whereas oxidation loss exceeds biosynthesis in old leaves. It also suggests that AsA content in sweet cherry leaves is controlled by developmental processes, which is consistent with what was found in apple leaves [37].

GalDH, GalLDH and GGP were key enzymes in L-galactose pathway of AsA synthesize [38, 39]. In the present study, the expression patterns of *GalDH*, *GalLDH* and *GGP* showed highest expression level in mature leaves, which was not good agreement with AsA content in leaves of different ages (Fig 5A and 5C). while three genes involved in recycling, MDHAR, DHAR1, and DHAR2, had extremely high expression levels in young leaves in contrast to mature and old leaves (Fig 5B), indicating their critical role in maintaining the high levels of AsA content in young leaves.

## Conclusions

AsA concentrations in plant cells are highly regulated by developmental processes such as fruit development. This regulation may differ according to genotype, tissue, and cell type. In the present study, we identified a complete and continuous set of sequences involved in the L-galactose pathway in AsA biosynthesis from sweet cherry fruit. The AsA content, expression, and activity levels of the corresponding proteins were detected during fruit development. AsA concentration was correlated with mRNA expression levels of *GGP2*, *GalLDH*, *AO2*, *APX3*, *GR1*, and *DHAR1*, all of which were highest in young fruit and then decreased steadily until full maturity. This indicates that these genes are the main control points for AsA production. More investigations are thus needed to achieve a deeper understanding of the role of key regulatory sites in sweet cherry fruit AsA accumulation.

## Supporting information

S1 Appendix AsA content and related anzyme activities during sweet cherry fruit development. (XLSX)

S2 Appendix Expression level of genes involved in AsA metabolism during sweet cherry fruit development. (XLSX)

S3 Appendix Expression level of genes involved in AsA metabolism in young, mature and old leaves. (XLSX)
